# Supplementary material for: Understanding Supramolecular Assembly of Supercharged Proteins
Source: ACS Cent Sci. 2022 Sep 13;8(9):1350–61. doi: 10.1021/acscentsci.2c00730 (PMC9523778; doi:10.1021/acscentsci.2c00730)
Supplement: Supplementary file 2 — oc2c00730_si_002.pdf [file oc2c00730_si_002.pdf]

oc-2022-00730k.R1

Name: Peer Review Information for "Understanding Supramolecular Assembly of Supercharged Proteins"

First Round of Reviewer Comments

Reviewer: 1

Comments to the Author

This contribution to ACS Central Science from the Schroeder group describes a detailed study of the supramolecular assembly of highly charged proteins, referred to as supercharged proteins. Although this descriptor for proteins seems vague, it appears to be the norm for the field. The work involves both experimental and computational modeling, and the conclusions are well justified by the experiments/modeling. They find that:

1) Minimally charged do not assemble into hierarchical assemblies,

2) The minimally and highly charged proteins are different even though the H-bonding and salt bridge possibilities are similar, thus revealing that long-range electrostatics are important in the fully charged proteins.

3) Specific inter-protein interactions are not sufficient to explain the assembly between oppositely charged proteins, thus implying that the rational design of protein interfaces should take into consideration the long-range interactions, not just local attractions.

4) The assembly is mediated by electrostatic screening by added salts and pH in a manner that would be expected, albeit assembly is guided by the distribution of the charges on the protein surfaces.

5) The results also demonstrate that proteins with similar net charges, but different distributions, will give rise to different hierarchical structures.

6) Finally, the kinetic stabilities (rates of exchange of assembly partners) depend upon the surface charge distribution.

Conclusions 1, 4 and 5 all seem pretty obvious, and would the investigators have expected anything different? Conclusions 2, 3 and 6 are less obvious, and are thus the most important guiding principles of the paper. This reviewer suggests that these less obvious, and/or others he/she may have missed, be more accentuated in the conclusions. Additionally, the primary focus of the introduction to the paper is on previous advances and strategies for protein assembly, with only a passing mention of mimicking natural biological assemblies, and the creation of drug delivery methods, sensors, and facilitated charge transport. These drivers for the study are vague, and the lessons from the studies that are directly applicable to these applications are lacking. The authors would be well served to beef up the reasons for the study in the introduction, and then return to these in the conclusions. Lastly, Figure 2d and 5d

are so dark, that the reader really cannot see what is being taught, and is essence must just rely on what the authors are saying. It would behove the authors to improve these images.

Reviewer: 2

Comments to the Author

This paper presents new studies on differently charged protein mutants to better understand the assembly of supercharged proteins into discrete nanomaterials. Careful and targeted protein design created different mutants with variable charge state and variable charge distribution for a given charge state. The assembly was explored using a variety of experimental and computational tools, mainly focusing on FRET efficiencies and particle size as a function of ionic strength, and MD simulations to extract energetics of different protein binding interfaces. A screen of different charge levels was explicated, but the results were not really unexpected. More interesting in my view is the study of different surface charge distribution, which is complicated to understand. Indeed, the results were not conclusive, with the main conclusion being “these results highlight the role of surface charge distribution” without giving any more specifics. Although the present work certainly does not clarify all aspects of supercharged protein assembly, it does provide some design concepts and highlights which interactions might be most influential in dictating higher order assembly of charged proteins. This manuscript seems to be of broad interest to a wide community of researchers, and I would recommend publication subject to addressing the revisions/comments below.

1) In the protein design and expression, they state the level of charging in the minimal case, but it is not explicit that _all_ the charges maintained on the minimal mutant are only those associated with inter-protein association in the 16-mers from the “wild type mutants” I assume this is the case based on their statement “they retain the requisite set of mutated amino acids involved in stabilizing the inter-protein interfaces in the 16-mer assembled structure”, but this does not discount the possibility of a couple extra charges beyond that requisite for the association.

2) Consistent qualitative comparisons are needed between the different analytical methods describing the impact of ionic strength (p 6-7). For example, in the FRET section, it is claimed “the minimally mutated variants do not associate as strongly as the supercharged protein pairs” (which could imply that both have some strong association?) while in the DLS it is stated “that Ceru-min and GFP-min do not strongly associate at the range of salt concentrations considered here.” I interpret this as some existence of interaction in FRET measurement that is not recorded in DLS?

3) Authors should speculate more on why the mutants do not display assembly as observed by TEM. Is there something specific about the mutant structure (e.g. placement of uncharged mutated residues) that effectively frustrates evaporative assembly? I would not necessarily expect the mutant to assemble into the organized 16-mer structure, but am surprised that _something_ is not formed beyond isolated proteins, especially for something that is poised to assemble into organized super-structures.

4) Related to above, they state “externally facing, non-interacting mutated charged amino acids are necessary to promote assembly” … at first I thought the word “mutant” should be removed here because the mutated protein does not assemble. But then I realized that they are comparing a mutant (supercharged protein) with a mutant of a mutant (the minimal charged structure). I would suggest to

more clearly and quickly distinguish between these two colloquially in text, perhaps with consistent use of "fully mutated" and "minimally mutated"?

5) The discussion of metastable states (p 12) seemed too speculative, and in fact, a bit generic for any type of assembly mechanism. The rest of their MD discussion is on much more quantitative footing and can be correlated to some extent to experiment or observation. If this does not advance their discussion, does it need to be included?

6) In the dynamic exchange section, more justification is needed for using dilution and subsequent FRET efficiencies as a metric for dynamic exchange. Any supramolecular complex is in equilibrium with unassembled units, and dilution drives this equilibrium to disassembly. It is therefore not unexpected that FRET ratios will decrease with dilution, or for greater charged components, that they were more resistant to the dissolution. It seems more appropriate as the authors do later to have a competitive protein with a unique spectral or spin signature that can be interrogated over time to reflect kinetics of subunit exchange in a given complex at a given solution condition.

Author's Response to Peer Review Comments:

Please see attached response letter. Thank you.

3247 Beckman Institute
405 N. Mathews Avenue
Urbana, IL 61801-3602 USA
Email: cms@illinois.edu
Phone: (217) 333-3906

Charles M. Schroeder III
James Economy Professor

To: Professor Editor
Senior Editor, *ACS Central Science*

Re: Response to reviewers for manuscript oc-2022-00730k

Date: August 15, 2022

Dear Dr. Editor,

I am writing to provide a revised manuscript and response to the reviewers for our manuscript **oc-2022-00730k** entitled "Understanding Supramolecular Assembly of Supercharged Proteins" that is being considered for publication in *ACS Central Science*. In this letter, we provide a detailed point-by-point response to the comments from two independent reviewers.

We sincerely thank the reviewers for providing insightful and constructive comments on our work. We believe that the reviewers' suggestions have improved the quality and clarity of the manuscript.

Based on the reviews, we extensively revised the main text and figures (**shown in red in the 'marked' version** of the manuscript).

In closing, we thank you for your kind consideration of our manuscript, and we look forward to hearing back. Please feel free to contact me if you have any questions.

Sincerely,

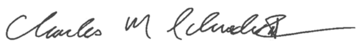

Charles M. Schroeder
James Economy Professor of Materials Science and Engineering
Professor, Department of Chemical & Biomolecular Engineering
Co-Chair, Molecular Science and Engineering Research Theme
Beckman Institute for Advanced Science and Technology
Department of Chemistry, *Affiliate*
Department of Bioengineering, *Affiliate*
Materials Research Laboratory (MRL), *Affiliate*
Center for Biophysics and Quantitative Biology
Carl R. Woese Institute for Genomic Biology, *Affiliate*
University of Illinois at Urbana-Champaign

Responses to the Reviewers' Comments
We are grateful to the reviewers for their constructive comments on our manuscript. The reviewers' suggestions provided a deeper understanding of the results and enhanced the quality of the manuscript. In the following section, we address all of the reviewers' comments in a point-by-point manner and we describe the specific changes/additions/corrections made to the manuscript based on these comments. Reviewer comments are listed in *italics,* while our responses follow in plain text, and changes/additions to the manuscript text are shown in as "quoted text".

Response to Reviewer #1:

Overall comment: *This contribution to ACS Central Science from the Schroeder group describes a detailed study of the supramolecular assembly of highly charged proteins, referred to as supercharged proteins. Although this descriptor for proteins seems vague, it appears to be the norm for the field. The work involves both experimental and computational modeling, and the conclusions are well justified by the experiments/modeling. They find that:*

1) Minimally charged do not assemble into hierarchical assemblies.
2) The minimally and highly charged proteins are different even though the H-bonding and salt bridge possibilities are similar, thus revealing that long-range electrostatics are important in the fully charged proteins.
3) Specific inter-protein interactions are not sufficient to explain the assembly between oppositely charged proteins, thus implying that the rational design of protein interfaces should take into consideration the long-range interactions, not just local attractions.
4) The assembly is mediated by electrostatic screening by added salts and pH in a manner that would be expected, albeit assembly is guided by the distribution of the charges on the protein surfaces.
5) The results also demonstrate that proteins with similar net charges, but different distributions, will give rise to different hierarchical structures.
6) Finally, the kinetic stabilities (rates of exchange of assembly partners) depend upon the surface charge distribution.

Response: We sincerely thank the reviewer for the thoughtful comments. Based on these suggestions, we revised the manuscript to improve the clarity on the key conclusions, as discussed below.

Comment 1: *Conclusions 1, 4 and 5 all seem pretty obvious, and would the investigators have expected anything different? Conclusions 2, 3 and 6 are less obvious, and are thus the most important guiding principles of the paper. This reviewer suggests that these less obvious, and/or others he/she may have missed, be more accentuated in the conclusions.*

Response 1: The reviewer has succinctly identified the main conclusions of the paper. We agree that conclusions 2, 3, and 6 were not obvious. Nevertheless, some of the other (seemingly obvious) conclusions were indeed surprising to us. For example, regarding conclusion 1, we initially expected to observe some amount of assembly between the minimally mutated variants because they retained the interfaces necessary to generate the 16-mer assembled structure. Upon observing no assembly for the minimally mutated variants, we turned to computational modeling to understand the assembly process for these proteins. Regarding conclusion 4, we were surprised by the observation that net charge is a sufficient descriptor to predict whether supercharged proteins assemble macroscopically at different solution conditions, even for proteins with significantly different surface charge distributions. We had not expected to observe a general trend in behavior using a minimal descriptor of the system. Regarding

conclusion 5, observing different hierarchical assembled structures from different surface charge distributions was less surprising, and as such, these results occupy less space in the main text.

To address these comments, we revised the conclusion section of the main text to highlight and discuss the less obvious conclusions noted by the reviewer.

Revised text: p. 24 "Unexpectedly, ΔG^{0}_{bind} for the minimally charged protein variants are significantly smaller (~30%) than those of the fully charged variants even though the interfaces retain all specific local attractions (e.g., H-bonding and salt bridge interactions)."

p. 24 "Furthermore, our results suggest that identification of specific inter-protein interactions from high resolution structures alone is insufficient to fully describe the interactions and assembly process between oppositely supercharged proteins. Our results show that assembly of supercharged proteins is governed by both these local attractions as well as long-range electrostatic interactions. Therefore, these findings will be useful for informing the rational design of new hierarchical structures based on predictions of complementary surfaces.[73-77]"

p. 25 "Surprisingly, the kinetic stabilities of these different supramolecular structures were found to depend on surface charge distribution, suggesting that assembled structures with different functional properties can be formed from a common building block."

Comment 2: *Additionally, the primary focus of the introduction to the paper is on previous advances and strategies for protein assembly, with only a passing mention of mimicking natural biological assemblies, and the creation of drug delivery methods, sensors, and facilitated charge transport. These drivers for the study are vague, and the lessons from the studies that are directly applicable to these applications are lacking. The authors would be well served to beef up the reasons for the study in the introduction, and then return to these in the conclusions.*

Response 2: To address this comment, we revised the introduction to emphasize the primary drivers and applications that motivate our work. In particular, the text has been revised to emphasize that efficient discovery new biomaterials with novel function is only possible with a detailed, fundamental understanding of the protein assembly process.

Modified text: p. 2 "However, despite recent progress, the fundamental mechanisms governing biomolecular assembly are complex not yet fully understood, which has led to the construction of synthetic biomolecular assemblies using informed design strategies and trial and error experimentation. Overall, the development of new functional biomaterials would greatly benefit from achieving a detailed, fundamental understanding of the protein assembly process."

p. 3 "Given the complexity of biomolecular interfaces and interactions, many supramolecular assembly strategies are not easily generalizable across different classes of proteins, which limits their potential use in developing new functional biological materials."

p. 3 "Although electrostatic interactions provide a promising method of protein supramolecular assembly that could yield synthetic biological assemblies and materials with functional properties, we lack a complete understanding of the underlying assembly process and the molecular design rules governing electrostatic-mediated protein-protein interactions."

p. 5 "Overall, our work provides an improved understanding of the fundamental mechanisms by which oppositely supercharged proteins assemble, and these results will be useful in guiding the rational design of new synthetic biological assemblies."

Comment 3: *Lastly, Figure 2d and 5d are so dark, that the reader really cannot see what is being taught, and is essence must just rely on what the authors are saying. It would behove the authors to improve these images.*

Response 3: We thank the reviewer for these comments. To address these comments, we revised Figure 2d and Figure 5d to increase the size of the TEM images and enhance the image contrast, and we exported the figures at a higher resolution to improve the overall quality of the figures.

Response to Reviewer #2:

Overall comment: *This paper presents new studies on differently charged protein mutants to better understand the assembly of supercharged proteins into discrete nanomaterials. Careful and targeted protein design created different mutants with variable charge state and variable charge distribution for a given charge state. The assembly was explored using a variety of experimental and computational tools, mainly focusing on FRET efficiencies and particle size as a function of ionic strength, and MD simulations to extract energetics of different protein binding interfaces. A screen of different charge levels was explicated, but the results were not really unexpected. More interesting in my view is the study of different surface charge distribution, which is complicated to understand. Indeed, the results were not conclusive, with the main conclusion being "these results highlight the role of surface charge distribution" without giving any more specifics. Although the present work certainly does not clarify all aspects of supercharged protein assembly, it does provide some design concepts and highlights which interactions might be most influential in dictating higher order assembly of charged proteins. This manuscript seems to be of broad interest to a wide community of researchers, and I would recommend publication subject to addressing the revisions/comments below.*

Response: We thank the reviewer for the insightful comments and review of our manuscript.

Comment 1: *In the protein design and expression, they state the level of charging in the minimal case, but it is not explicit that _all_ the charges maintained on the minimal mutant are only those associated with inter-protein association in the 16-mers from the "wild type mutants". I assume this is the case based on their statement "they retain the requisite set of mutated amino acids involved in stabilizing the inter-protein interfaces in the 16-mer assembled structure", but this does not discount the possibility of a couple extra charges beyond that requisite for the association.*

Response 1: The reviewer is correct in that the only mutated amino acids retained in the minimal mutants (relative to "wild type") are those previously identified as participating in protein-protein interfaces of the assembled 16-mer structure. We revised the text to be very explicit on this point.

Modified text: p. 6 "To understand the role of non-interacting charged residues in supramolecular assembly of supercharged proteins, we designed and expressed two minimally mutated GFP and Cerulean variants that contain only the mutations participating in inter-protein interactions and do not contain any of the externally facing, non-interacting charged amino acids identified by Simon et al."

Comment 2: *Consistent qualitative comparisons are needed between the different analytical methods describing the impact of ionic strength (p 6-7). For example, in the FRET section, it is claimed "the minimally mutated variants do not associate as strongly as the supercharged protein pairs" (which could imply that both have some strong association?) while in the DLS it is stated "that Ceru-min and GFP-min do not strongly associate at the range of salt concentrations considered here." I interpret this as some existence of interaction in FRET measurement that is not recorded in DLS?*

Response 2: The reviewer is correct. In general, DLS is a less sensitive probe of association than FRET measurements because it only provides average size information on the dominant species in solution. FRET measurements provide information regarding the average nanoscale (~1-10 nm) distance between proteins, which is useful in informing on potential protein interactions (albeit without structural insight). Here, our results show that FRET efficiency between the minimally mutated variants is <0.5 at all salt concentrations, suggesting that the proteins are (on average) separated by a distance larger than the Förster length (5.5 nm between GFP and Cerulean) at all salt concentrations. This distance is significantly larger than the separation distance observed in the assembled 16-mer structure (~4 nm), indicating weak association. Thus, both the FRET and DLS measurements indicate that the minimally mutated variants are weakly associated at low salt concentrations. To address this comment, we revised the main text to address these points more consistently.

Modified text: p. 7 "Our results show that the FRET efficiencies between Ceru-min and GFP-min are significantly lower than those between the fully supercharged variants at all NaCl concentrations and are < 0.5 at all salt concentrations, indicating weakly associating proteins. Ultimately, these results suggest that minimally mutated variants do not associate as strongly as the supercharged protein pairs."

Comment 3: *Authors should speculate more on why the mutants do not display assembly as observed by TEM. Is there something specific about the mutant structure (e.g. placement of uncharged mutated residues) that effectively frustrates evaporative assembly? I would not necessarily expect the mutant to assemble into the organized 16-mer structure, but am surprised that _something_ is not formed beyond isolated proteins, especially for something that is poised to assemble into organized super-structures.*

Response 3: We agree with the reviewer in that we were also surprised by the lack of assembly between the minimally mutated variants. Indeed, these experimental results motivated us to pursue computational modeling to understand the role of electrostatic interactions on protein assembly. Results from MD simulations showed that the non-interacting amino acids away from the protein-protein interface stabilize assemblies via long-range electrostatic interactions and are required to nucleate assembly between the proteins. To address this comment, we revised the main text to better connect the experimental observations and the MD simulations.

Modified text: p. 8 "Based on the lack of assembly between the minimally mutated variants, we hypothesized that the non-interacting mutated charged amino acids promote assembly by two potential mechanisms: (1) the non-interacting surface charges contribute stabilizing long-range electrostatic interactions within the assembled structure, or (2) the non-interacting surface charges are required to nucleate protomer formation. To test the validity of these hypotheses, atomistic MD simulations were performed…"

Comment 4: *Related to above, they state "externally facing, non-interacting mutated charged amino acids are necessary to promote assembly" ... at first I thought the word "mutant" should be removed*

here because the mutated protein does not assemble. But then I realized that they are comparing a mutant (supercharged protein) with a mutant of a mutant (the minimal charged structure). I would suggest to more clearly and quickly distinguish between these two colloquially in text, perhaps with consistent use of "fully mutated" and "minimally mutated"?

Response 4: We thank the reviewer identifying this ambiguity. To address this comment, we revised the text to enhance clarity.

Modified text: p. 8 "Thus, our results show that the minimally mutated variants are incapable of forming the ordered 16-mer structure, and the externally facing, non-interacting charged amino acids in the fully charged variants are necessary to promote assembly."

Comment 5: *The discussion of metastable states (p 12) seemed too speculative, and in fact, a bit generic for any type of assembly mechanism. The rest of their MD discussion is on much more quantitative footing and can be correlated to some extent to experiment or observation. If this does not advance their discussion, does it need to be included?*

Response 5: We removed the brief discussion of metastable states. The following text was removed:

p. 12 "~~Overall, the process of protein association is highly non-specific, and a continuum of metastable states exist in which the transiently formed interfaces do not lead to the final interface observed in the assembled structures. Such metastable states are accessible in the absence of a dominant free-energy minima that overpowers the preference for a single interface. We posit that these metastable states become more accessible in the minimally mutated variants, and hence result in non-specific binding in minimally charged protein dimers.~~"

Comment 6: *In the dynamic exchange section, more justification is needed for using dilution and subsequent FRET efficiencies as a metric for dynamic exchange. Any supramolecular complex is in equilibrium with unassembled units, and dilution drives this equilibrium to disassembly. It is therefore not unexpected that FRET ratios will decrease with dilution, or for greater charged components, that they were more resistant to the dissolution. It seems more appropriate as the authors do later to have a competitive protein with a unique spectral or spin signature that can be interrogated over time to reflect kinetics of subunit exchange in a given complex at a given solution condition.*

Response 6: We agree that dilution shifts equilibrium to disassembly, and as expected, this leads to decreasing FRET ratios. However, we note that the dilution experiments were simply merely used to provide a qualitative metric for which protein combinations showed slow dynamic subunit exchange (>15 minutes), and therefore informed on potential candidates to study using the non-fluorescent protein analogs. We emphasize that no quantitative dynamic exchange kinetics were measured using dilution in this study. To address this point, we revised the main text to clarify that dilution experiments were only used to provide qualitative information on protein exchange.

Modified text: p.19 "First, the concentration dependence of supramolecular assemblies was assessed using dilution experiments to qualitatively assess kinetic stability protein assemblies. In these experiments, the assembled protein structures were diluted with buffered solution, and equilibrium FRET ratios were measured after ~15 minutes (**Figure S14**). We note that these dilution experiments were only used to identify potential candidates for dynamic subunit exchange experiments. If proteins rapidly exchange with nearby protein partners in solution…"
